# Effects of population outcrossing on rotifer fitness

**DOI:** 10.1186/1471-2148-10-312

**Published:** 2010-10-18

**Authors:** Ana M Tortajada, María José Carmona, Manuel Serra

**Affiliations:** 1 Institut Cavanilles de Biodiversitat i Biologia Evolutiva, Universitat de València, A.O. 22085, València 46071, Spain

## Abstract

**Background:**

Outcrossing between populations can exert either positive or negative effects on offspring fitness. Cyclically parthenogenetic rotifers, like other continental zooplankters, show high genetic differentiation despite their high potential for passive dispersal. Within this context, the effects of outcrossing may be relevant in modulating gene flow between populations through selection for or against interpopulation hybrids. Nevertheless, these effects remain practically unexplored in rotifers. Here, the consequences of outcrossing on the rotifer *Brachionus plicatilis *were investigated. Cross-mating experiments were performed between a reference population and three alternative populations that differed in their genetic distance with regard to the former. Two offspring generations were obtained: F1 and BC ('backcross'). Fitness of the outcrossed offspring was compared with fitness of the offspring of the reference population for both generations and for three different between-population combinations. Four fitness components were measured throughout the rotifer life cycle: the diapausing egg-hatching proportion, clone viability (for the clones originating from diapausing eggs), initial net growth rate *R *for each viable clone, and the proportion of male-producing clones. Additionally, both the parental fertilisation proportion and a compound fitness measure, integrating the complete life cycle, were estimated.

**Results:**

In the F1 generation, hybrid vigour was detected for the diapausing egg-hatching proportion, while *R *was lower in the outcrossed offspring than in the offspring of the reference population. Despite these contrasting results, hybrid vigour was globally observed for the compound measure of fitness. Moreover, there was evidence that this vigour could increase with the genetic differentiation of the outcrossed populations. In the BC generation, the hybrid vigour detected for the egg-hatching proportion in the F1 generation reverted to outbreeding depression. By contrast, signs of hybrid vigour were observed for clone viability and *R*. The opposing trends observed for different life-cycle stages yielded a global pattern of hybrid vigour in the BC generation for two out of the three between-population comparisons.

**Conclusions:**

Results suggest that outbreeding depression does not constitute a barrier to gene flow. In newly-founded populations, where the population size is still small, dilution of immigrants should be low. Thus, a lack of outbreeding depression would allow gene flow to have an impact on the genetic composition of these populations.

## Background

The effects of inbreeding arising from non-random mating or random mating in small populations have been widely explored, and an inbreeding depression of fitness has often been documented for a great variety of organisms [see e.g. [1]]. By contrast, the effects of outbreeding resulting from mating between individuals originating from different locations that can vary in their degree of genetic relatedness have been analysed to a lesser extent [2]. When individuals from two populations mate, either positive or negative consequences can arise for offspring fitness [3,4]. Such unpredictable consequences of outbreeding result from complex genetic processes. According to Edmands' review [4], increased fitness, known as hybrid vigour, frequently follows outcrossing; this hybrid vigour is thought to be caused by dominance (masking of deleterious recessive alleles), overdominance or epistasis. Conversely, diminished fitness, known as outbreeding depression, can also arise. Outbreeding depression can appear as early as the F1 generation and is attributed to underdominance, disruption of local adaptation or epistatic interactions. However, this type of depression is more frequent in the F2 or in backcross generations, when the original parental gene combinations are disrupted [5].

The consequences of outcrossing can affect population genetic differentiation, since gene flow could either be promoted by hybrid vigour [6,7] or hindered by outbreeding depression [8]. The unexpected population genetic structure detected for continental zooplankton organisms such as cladocerans and rotifers turns this relationship into a subject worthy of exploration. High levels of genetic differentiation regarding neutral markers, as well as ecologically relevant traits, have been detected, even among nearby populations of cladocerans [9] and rotifers [10,11]. This differentiation is striking, since evidence for high passive dispersal ability via dormant propagules exists for these organisms [12]. De Meester *et al*. [13] extended the 'persistent founder effects' idea of Boileau *et al*. [14] to postulate the 'Monopolisation Hypothesis', which explains the unexpected genetic differentiation detected among populations of pond-dwelling organisms. According to this hypothesis, such differentiation is due to persistent founder effects, caused and maintained by a large population size reached rapidly from a few colonisers, and local adaptation in the descendants of these first colonisers. These mechanisms seem to be powerful enough to hinder the establishment of late-comers, i.e., to hamper gene flow and consequently to maintain genetic differentiation. Nevertheless, as mentioned above, outbreeding effects could also be relevant in shaping this genetic structure.

Rotifers are small invertebrates (ca. 50-2000 μm) commonly found in the water column of ponds and lakes [15]. Most rotifer species are cyclical parthenogens, combining sexual and asexual reproduction. Initially, a cyclically parthenogenetic rotifer population consists of only diploid asexual females, which produce asexual daughters by ameiotic parthenogenesis. Thus, the rotifer population is a set of clones. Production of sexual females is induced by environmental factors such as population density [16]. Rotifer sexual females produce haploid eggs that develop into haploid males, or, if fertilised by a male, into diapausing eggs (commonly called 'resting eggs' in the literature on rotifers), which can tolerate adverse environmental conditions. When sexual reproduction is initiated, it affects only a fraction of the population, so that parthenogenetic proliferation does not stop. Rotifer populations persist for part of the year as diapausing eggs in most places. A fraction of the diapausing eggs hatch into asexual diploid females when favourable conditions resume, and the rest remain viable in the sediment and form an 'egg bank' [17,18]. Cyclical parthenogenesis is thought to facilitate population differentiation via resource monopolisation because: (1) high population sizes are rapidly reached and maintained though asexual reproduction and the diapausing egg bank in the sediment; and (2) clonal selection during the phase of asexual reproduction and the release of hidden genetic variation through recurrent sexual reproduction enables the emergence of local adaptation [19].

Cyclical parthenogenesis can also affect the consequences of outbreeding between populations of the same species and, in turn, the effect of outcrossing on gene flow. Evidence for clonal selection has been detected in natural rotifer populations [20]. This could favour the formation of coadapted gene complexes or the appearance of local adaptation. Consequently, regardless of the relative contribution of other potentially involved genetic mechanisms, one would expect the appearance of negative epistatic effects after outcrossing, and thus an outbreeding depression of fitness hindering gene flow. An additional factor that could affect outbreeding effects in cyclical parthenogenetic rotifers is male haploidy, which is thought to favour the purging of deleterious recessive alleles from populations through haploid males [21,22], thereby diminishing the probability of inbreeding depression in rotifer populations and providing fewer advantages for outbreeding. Surprisingly, evidence for inbreeding depression has been detected for rotifers after selfing [23-25]. These findings indicate that the question as to whether hybrid vigour, rather than outbreeding depression, occurs in rotifers remains unanswered. This question is relevant to assess how population genetic differentiation is maintained. The uncertainty about outbreeding effects and the genetic structure detected in their populations makes cyclically parthenogenetic rotifers an excellent model to study population outcrossing effects and their consequences on gene flow.

**Figure 1:**
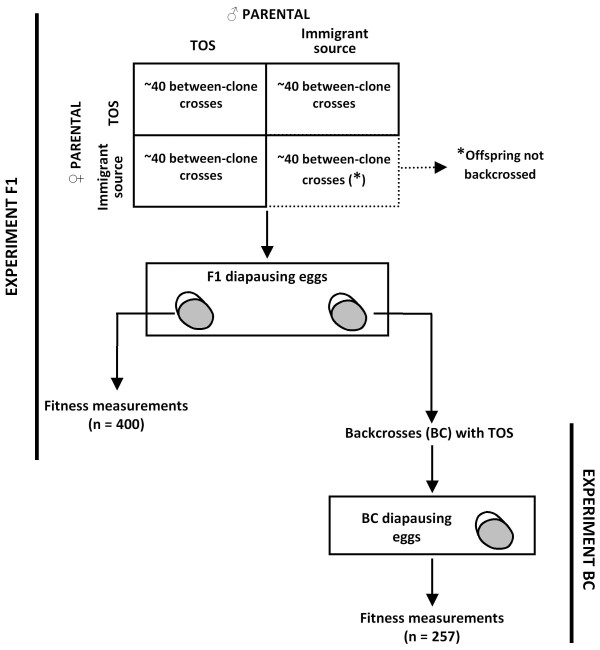
**Design of cross-mating experiments to obtain F1 and BC generations, performed with four *B. plicatilis *populations**. TOS population was regarded as a resident population and the other three (HOS, TUR and CHI) as alternative immigrant source populations. N is the number of eggs tested for hatching.

The cyclically parthenogenetic rotifer *Brachionus plicatilis *(Müller 1786) is a model species in ecological genetics and population ecology [e.g. [10,26]]. In this study, individuals of *B. plicatilis *from different populations were crossed to investigate the effect on offspring fitness. The objective was to shed light on the consequences of between-population outbreeding on gene flow. The experimental design tried to mimic the sequence of crosses that would be likely to occur in a large (not newly-founded) population after the arrival of immigrant individuals, so it focused on the crosses that would offer the opportunity for immigrant genes to be introduced into the resident population. Four *B. plicatilis *populations (acronyms: TOS, HOS, TUR and CHI; see Methods for details) were selected. TOS was regarded as a resident population where immigrants would arrive, and the other three were alternative immigrant source populations. Crosses between clones from these populations were conducted in the laboratory, and offspring fitness from intra- and interpopulation crosses was estimated by measuring life-history traits throughout the rotifer life cycle, from the diapausing-egg stage to the induction of sexual reproduction. Furthermore, the number of females obtained per incubated diapausing egg at the end of the experiment was used as a compound fitness measure. Additionally, the parental fertilisation proportion was estimated from the cross-mating experiments. As the negative effects of outbreeding often do not appear until the second generation [5], backcrosses were carried out to obtain a backcross (BC) generation. For backcrosses, two types of crosses were compared: (1) crosses between the F1 'resident × immigrant hybrids' and 'resident' clones (hereafter, hybrid-resident); and (2) crosses between the F1 'resident × resident' and 'resident' clones (hereafter, resident-resident; Figure 1). A clone was not used in more than one cross. The 'immigrant × immigrant' crosses were not tested, as they were assumed to be unlikely to occur in nature due to the dilution of immigrants in a large population of residents. Zooplankton mobility (i.e., vertical migration) and water column turbulence make spatial clustering of relatives improbable, thus reducing the chances of mating among the parthenogenetic offspring of an immigrant.

## Results

### F1 generation

**Figure 2:**
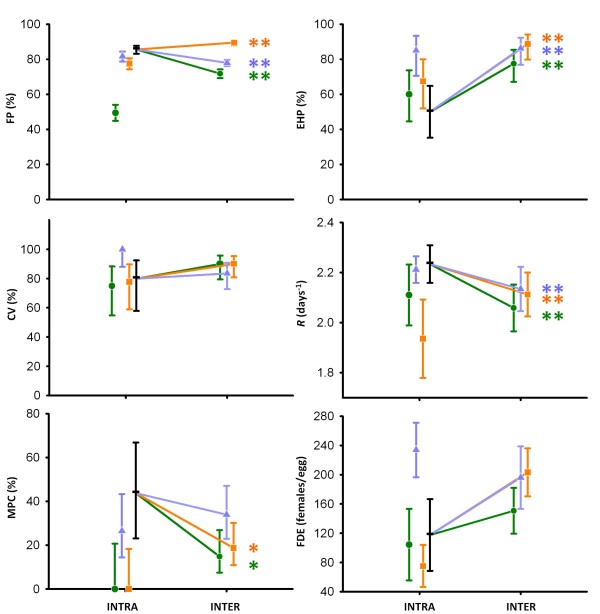
**Average values for the response in the F1 experiment, for intra- and interpopulation crosses**. Vertical bars are 95% confidence intervals; significant differences between interpopulation ('resident × immigrant') crosses and the intrapopulation ('resident × resident') cross: *: *P*-value < 0.05; **: *P*-value < 0.01 (colour code as below). Response in the intrapopulation 'immigrant × immigrant' crosses are also shown. Black horizontal bar, resident population (TOS); green circle, immigrant HOS; orange square, immigrant CHI; blue triangle, immigrant TUR. The values for the two interpopulation cross combinations are averaged. FP: fertilisation proportion; EHP: egg-hatching proportion; CV: clone viability; *R*: net growth rate of the viable F1 clones; MPC: proportion of male-producing clones; and FDE: number of females produced per incubated diapausing egg.

Significant effects of the type of cross ('resident × resident' *vs*. 'resident × immigrant') on the F1 generation fitness components were detected for the three between-population comparisons, i.e., TOS-HOS, TOS-TUR, and TOS-CHI. The type of cross affected the diapausing egg-hatching proportion and the initial net growth rate of the viable clones, *R*, for the three immigrant populations, and the proportion of male producing clones in two of the three comparisons. The diapausing egg-hatching proportion was positively affected by outbreeding. Evidence for hybrid vigour was found for TOS-HOS and TOS-CHI comparisons (Figure 2, EHP; see Additional file 1 for raw data), since the hatching proportion was significantly higher for F1 diapausing eggs produced in interpopulation crosses than for F1 diapausing eggs produced in both types of intrapopulation crosses (i.e., 'resident × resident' and 'immigrant × immigrant'). In the case of the TOS-TUR comparison, the hatching proportion of the eggs produced in interpopulation crosses was significantly higher than those produced in the intrapopulation crosses within the resident population, but it was very similar to the hatching rate of the immigrant offspring. In the case of the growth rate *R *for the three between-population cross comparisons, offspring of interpopulation crosses presented significantly lower average *R *values than offspring of intrapopulation crosses of the resident population (Figure 2, *R*). Interpopulation average *R *values were lower than immigrant average *R *values, except when CHI acted as immigrant source population (Figure 2).

No significant differences were observed in clone viability between 'resident × resident' and 'resident × immigrant' crosses in any of the three comparisons between populations. Moreover, there was no consistent trend suggesting either hybrid vigour or outbreeding depression (Figure 2; CV). The proportion of viable clones that were male-producing was lower in the outcrosses than in the intrapopulation crosses of the resident population; this effect was significant when CHI and HOS acted as immigrant populations (Figure 2; MPC). Nevertheless, interpopulation offspring always had values for the proportion of male-producing clones intermediate to those of immigrant and resident offspring.

Despite the diverse results among fitness components, the results for the compound measure of fitness (Figure 2, FDE) showed that offspring from interpopulation crosses produced a higher average number of females per incubated diapausing egg than the offspring from both types of intrapopulation crosses, except in the case of the TOS-TUR comparison where interpopulation offspring showed lower values than 'immigrant × immigrant' intrapopulation crosses offspring. To sum up, there was a global trend for hybrid vigour, except when TUR acted as the immigrant population. Interestingly, the compound measure of fitness ranked in the same order as the genetic distance between the outcrossed populations, as assessed by mitochondrial and microsatellite markers, such that an increase in genetic distance was associated with an increase in F1 fitness.

Results on the parental fertilisation proportion differed depending on the immigrant population. TOS-CHI interpopulation crosses had a significantly higher average fertilisation proportion than both intrapopulation crosses of the resident population and intrapopulation crosses of the immigrant population (Figure 2, FP). On the other hand, when TUR or HOS acted as immigrant populations, interpopulation crosses had a lower average fertilisation proportion than the intrapopulation crosses of the resident population. Nevertheless, values for the fertilisation proportion in interpopulation crosses where higher than in immigrant intrapopulation crosses. Finally, significant asymmetries in the fertilisation proportion, which depended on whether the immigrant clone was paternal or maternal in the cross combination, were detected for TOS-TUR and TOS-CHI comparisons (Table 1).

**Table 1:** Sex-dependent asymmetries in the fertilisation proportions.

Interpopulation cross	Generation	**Resident paternal × immigrant**^**a **^**maternal**	**Resident maternal × immigrant**^**a **^**paternal**	*P*-value
TOS-HOS	F1	74.7%	69.7%	0.057
	BC	91.6%	84.8%	<0.001
TOS-TUR	F1	73.3%	82.5%	<0.001
	BC	88.1%	84.0%	0.009
TOS-CHI	F1	92.6%	87.7%	0.002
	BC	85.6%	85.5%	0.951

*a*. For F1, clones used as immigrants were collected from the field; for BC, immigrant refers to F1 clones produced in 'immigrant × resident' crosses.

Parental fertilisation percentages and *P*-values for Chi-square tests applied to the three interpopulation crosses for F1 and BC generations.

### Backcross generation

**Figure 3:**
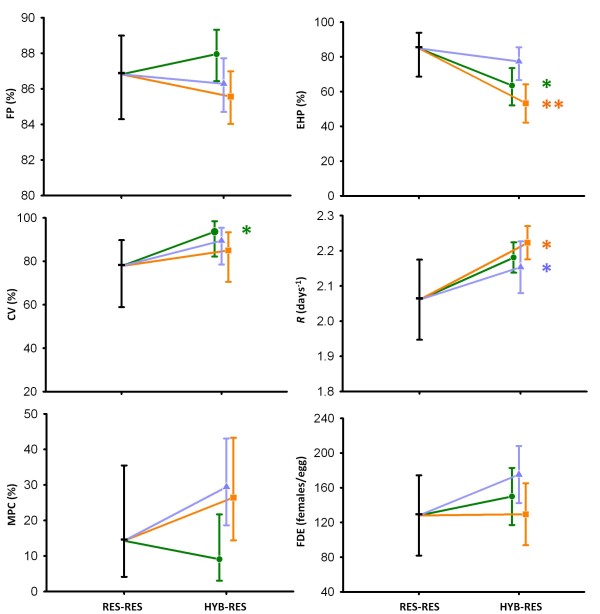
**Average values for the response in the BC experiment, for resident-resident and hybrid-resident crosses**. Vertical bars are 95% confidence intervals; significant differences between resident-resident and hybrid-resident crosses: *: *P*-value < 0.05; **: *P*-value < 0.01 (colour code as below). Black horizontal bar, BC for residents; green circle, BC for HOS as immigrant; orange square, BC for CHI as immigrant; blue triangle, BC for TUR as immigrant. FP: fertilisation proportion; EHP: egg-hatching proportion; CV: clone viability; *R*: net growth rate of the viable F1 clones; MPC: proportion of male-producing clones; and FDE: number of females produced per incubated diapausing egg.

In the BC generation, diapausing egg-hatching proportion, clone viability, and *R *-i.e., all the fitness components related to the asexual phase of the life cycle - were significantly affected by the type of cross ('resident-resident' *vs. *'hybrid-resident') in at least one of the three between-population cross comparisons (Figure 3; EHP, CV and *R*; see Additional file 2 for raw data), and the same trend was found regardless of the immigrant population. However, depending on the fitness components, either a trend to decreased or increased average values in the crosses involving immigrants was found. Diapausing egg-hatching proportion was lower in the backcrosses involving immigrants (Figure 3, EHP), while the opposite trend was observed for clone viability and *R *of the viable BC clones (Figure 3, CV and *R*).

Results for the compound measure of fitness (Figure 3, FDE) indicate a global tendency towards higher fitness in the backcrosses involving immigrants in two of the three between-population cross comparisons, while in one case (TOS-CHI) the mean value for this trait was virtually the same for resident-resident and hybrid-resident offspring. In contrast to the F1 generation, in the backcrosses the compound measure of fitness did not rank with the genetic distance between the outcrossed populations.

Concerning the proportion of viable male-producing clones, a fitness component related to the sexual phase of the life cycle, there were no significant differences between BC clones produced from resident-resident and hybrid-resident crosses (Figure 3, MPC). Likewise, no significant results were found for the BC fertilisation proportion (Figure 3, FP). Nevertheless, the analysis of differences depending on whether the resident was the paternal or the maternal clone revealed that, when TUR and HOS acted as immigrant populations, there were significant asymmetries (see Table 1).

## Discussion

How population differentiation occurs and how new species arise in invertebrates with high dispersal capability are crucial evolutionary questions. Application of molecular markers to aquatic invertebrates is showing previously unnoticed levels of species richness and population divergence. For aquatic invertebrates with high dispersal capability, knowing the fate of immigrants arriving in a population is highly relevant to understanding how populations maintain their genetic differences. Even though they are largely constrained to living in ponds and lakes, rotifers have been successful at diversifying in order to occupy a wide range of ecological niches. Within-species differentiation in rotifers, sometimes associated with local adaptation, has been reported [11]. Population differentiation in rotifers, likely a necessary step leading to speciation, might be promoted or prevented depending on the effects of outcrossing on fitness. However, in spite of its relevance, data on these effects are extremely scarce for continental zooplankters, particularly for rotifers [24,27]. In contrast to the previous studies on the phylum Rotifera, the present study adopts a population approach by exploring a high number of clones collected in four populations with known genetic distances [10,11]. Moreover, outcrossing effects are studied by: (1) performing controlled cross-mating experiments to obtain two generations with a well-known rotifer species; and (2) analysing offspring fitness throughout the full life cycle.

Our results suggest that outcrossing between a resident population and three alternative immigrant source populations of the rotifer *B. plicatilis *resulted in hybrid vigour in the F1 generation. A trend towards hybrid vigour was observed regardless of the immigrant source population, according to the results for the compound fitness measure. Nevertheless, we also found differences in the performance of the within-population offspring among the four experimental populations. These differences imply that the outcrossing effects act in opposite directions, depending on which population is regarded as immigrant and which as resident. This ambivalence appears in the F1 generation for the compound fitness component in the TOS-TUR comparison: the outcrossed offspring had higher fitness (i.e., produced more females per incubated diapausing egg) than the offspring from residents, but not than the offspring from crosses between immigrants. As it can be observed in Figure 2, experimental conditions, which were intended to be close to real TOS habitat conditions, were especially favourable for TUR, since within-population offspring for this pond presented very high values for this fitness component. This caveat on ambivalent results does not affect the other immigrant populations. The hybrid vigour observed in the F1 generation for the compound measure of fitness seems to be largely due to the effect of the hatching proportion, since these two parameters showed similar patterns, which is not true for other fitness components. When compared to the offspring produced by clones from TOS, the higher the genetic distance between populations, the higher compound fitness measure of the offspring. Since only four populations are compared, the hypothesis of a relationship between genetic distance and hybrid vigour in the F1 generation should be considered preliminary. Signs of hybrid vigour persisted in the backcrosses between the F1 hybrids and the resident population, except when CHI acted as the immigrant source population. However, lack of backcrosses between 'immigrant' and 'immigrant-resident hybrids' makes it more difficult for this finding to be conclusive.

Hybrid vigour in the F1 generation is generally expected after population outcrossing [3,28], and examples for a great variety of taxa abound in the literature [e.g. [29-31]], although the reverse result has also been found [e.g. [32,33]]. By contrast, outbreeding depression is usually expected in subsequent generations [3,28], or in the offspring of backcrosses. However, the majority of studies on outbreeding effects addresses a single generation [4,34], leaving open the question as to how these effects could affect gene flow between populations.

A zooplankter where outcrossing has been extensively studied is the marine copepod species *Tigriopus californicus*. In this invertebrate, outcrossing effects over several generations--including backcrosses--have been widely studied [e.g. [30]], in addition to its underlying genetic basis [e.g. [35-37]]. In general, these studies reveal hybrid vigour in the F1 generation, but hybrid breakdown in the F2 and BC generations. However, Edmands et al. [38] suggested that, if the offspring from backcrosses were allowed to reproduce over several generations, hybrid inferiority could change to hybrid superiority in later generations. Aside from copepods, two other common groups in the continental zooplankton are rotifers and cladocerans. These groups share features of their life cycles, as most of them are cyclical parthenogens, but they differ in their maximum growth rate, generation time, and maximum population density and size. They also differ because cladoceran males are not haploid, although the role of rotifer haploid males in purging recessive deleterious alleles is likely unimportant [23,24]. These similarities and differences make comparison between rotifers and cladocerans interesting. Using the cladoceran *Daphnia magna*, which is a cyclical parthenogen, Ebert et al. [7] performed a study using an experimental-field metapopulation that showed a trend towards hybrid vigour, as estimated from genotype frequency change. In contrast, De Meester [39] found negative outbreeding effects on the diapausing egg-hatching proportion for *D. magna *in a laboratory study on inbreeding and outbreeding depression. The hybrid vigour exhibited by cladocerans observed by Ebert *et al. *[7] is in agreement with the results of the present study for the compound measure of fitness. It is noteworthy that the local cladoceran populations studied in Ebert *et al. *[7] were highly inbred. By contrast, inbreeding does not seem to be important in the rotifer populations considered here. Evidence for inbreeding by non-random mating has not been found [10], while inbreeding by drift is unlikely in the typically large, genetically diverse populations studied here [20]. According to the results of the present study, outbreeding depression can be found in some fitness components, while hybrid vigour is detected if assessed by the compound measure of fitness. This finding may help to explain the contrasting results found in *Daphnia*.

The few studies dealing with the effects of population outcrossing on rotifers [24,27] used different experimental schemes, making comparisons with the present work difficult. For example, Birky [24] compared the viability of offspring by crossing eight clones from two geographically distant populations and selfish offspring. This author found no significant difference, which could be in agreement with the absence of a significant and consistent pattern of outbreeding effects for F1 viability in the present study. Given the scarcity of data on outbreeding effects in zooplankters, generalisations are difficult. The effects might be very dependent on the system studied, for instance on the age of the population. Moreover, laboratory conditions are expected to be more benign compared to conditions in the wild, although, qualitatively, the relative effects of outcrossing *vs*. within-population crosses are likely to hold under natural conditions.

The results of this study highlight that opposing effects are possible for the same fitness component when different sexual generations are compared. This was the case for the diapausing egg-hatching proportion--the first step of life cycle in which the fitness of a cross was measured--and the net growth rate. The pattern found for the diapausing-egg hatching proportion is coincident with the theoretical expectation of F1 hybrid vigour followed by hybrid breakdown in subsequent outcrossing events [3,28]. It is common to find either inbreeding or outbreeding effects in those fitness components related to the initial phases of the life cycle. Often the effects are observed in connection with embryo development [examples with different taxa: [32,39-41]]. Such effects seem very likely to be due to the high quantity of genes expressed during development. Concerning the net growth rate, the opposite patterns between F1 and BC generations found here suggest outbreeding depression in the F1 generation, followed by hybrid vigour in the BC generation. Outbreeding depression in F1 can be explained by a disruption of local adaptation or epistatic interactions between heterozygote loci [4]. A positive effect of outbreeding on the growth rate in the backcrosses could be explained if low-fitness hybrids are eliminated at the beginning of the life cycle (i.e., diapausing eggs do not hatch). Hatchlings from the diapausing eggs are expected to harbour high genetic diversity, since they are produced sexually, and some of them could have high fitness. Nevertheless, in both F1 and BC generations, the compound measure of fitness suggests advantages to the hybrid offspring produced from immigrants coming from some populations.

Our results on fertilisation proportions do not suggest outbreeding avoidance. In F1, in one case (immigrant: HOS) fertilisation was much lower between immigrant and resident than between residents, but it can be explained by the lower fertilisation proportion between immigrants. Asymmetry between the sexes was detected for the fertilisation proportion in crosses performed to produce the F1 and BC generations. In the F1 generation, gene flow is not consistently preferentially mediated by a sex. By contrast, in the BC generation gene flow is mainly mediated by females where significant asymmetries were found. Given the few populations analysed, our results do not consistently support a preference in the sex mediating gene flow.

Results on outbreeding effects showed some variation with regard to the populations crossed. This could be due to the genetic distance between the populations crossed. The relationship between the compound fitness measure at F1 and the genetic distance between populations, even based on few populations, is suggestive. Another factor that could contribute to the differences in the results for the three between-population comparisons concerns the strength of the negative inbreeding effects within the outcrossed populations. A previous study with TOS and HOS populations showed that inbreeding depression occurred at the population level for some of the life-history traits studied here [23], which is in accordance with the pattern of hybrid vigour detected here for these populations.

Cyclical parthenogenesis is expected to facilitate the development of coadapted gene complexes because selection acts on the total genetic variance during the parthenogenetic phase. In fact, clonal selection has been reported in rotifers [20]. If coadapted gene complexes are frequent, outbreeding depression is likely to occur. However, our results are not consistent with this prediction. This discrepancy can be explained if ecological differentiation between rotifer populations is weak. It has been proposed that temporal fluctuations can prevent high ecological specialisation in *Brachionus *populations [11]. Moreover, our results suggest that the negative effects of outbreeding arising in post-F1 generations could be purged in the first steps of the life cycle, while genetic variance from the outcrosses would allow selection of high-fitness genotypes. If so, outbreeding depression would not be important in maintaining genetic differentiation.

## Conclusions

This study focuses on the consequences of outcrossing between populations of the cyclically parthenogenetic rotifer *B. plicatilis*. This is relevant to assessing the mechanisms that maintain among-population genetic differentiation, which is high despite the high passive dispersal capability of these organisms. In the first outcrossed generation, some fitness components associated with different life-cycle stages were significantly affected, with the overall effect of outcrossing being positive. Backcrosses resulted in lower diapausing (sexual) egg hatchability. However, the clones founded from these hatchings showed high fitness. We found that the higher the genetic distance between immigrant and resident population, the higher the compound measure of fitness in the hybrid F1. A general conclusion of our study is that outbreeding depression is not likely in the studied populations. This conclusion seems to rule out outbreeding depression as a key factor maintaining population genetic differentiation. Rather, hybrid vigour opens up opportunities for gene flow into recently founded, small rotifer populations, where the dilution effect of immigrants would not be strong yet.

## Methods

### Source populations

*B. plicatilis *populations from four ponds in Eastern Spain were sampled: Poza Sur de Torreblanca (TOS) in 'Prat de Cabanes-Torreblanca' Nature Reserve; Hondo Sur (HOS) in 'El Hondo de Elche' Nature Reserve; Estany d'en Túries (TUR) in 'Els Aiguamolls de l'Empordà' Nature Reserve; and Salada de Chiprana (CHI), which belongs to the pond complex 'Saladas de Chiprana', located in the Ebro river basin. TOS, TUR, and HOS are coastal ponds, whereas CHI is an inland pond. TOS and HOS were also used in a previous study on inbreeding depression at the population level [23]. Moreover, the four selected populations had previously been used in phylogeographical and population genetic studies [10,11]. TOS was regarded as the resident population, and the genetic distances between TOS and the other source populations for immigrants were: F_ST _= 0.20, Φ_ST _= 0.47; F_ST _= 0.23, Φ_ST _= 0.67; and F_ST _= 0.51, Φ_ST _= 0.71 for HOS, TUR, and CHI, respectively (F_ST _values based on seven microsatellite loci and computed using Genetix 4.05 [42] from data in [11] and raw data used in [10], provided by A. Gómez [10]; Φ_ST _values based on cytochrome oxidase subunit I sequences [11]). Rotifers were sampled as diapausing eggs from superficial sediment samples from the ponds. *B. plicatilis *diapausing egg banks had been detected in the sediment of these ponds in preceding studies [18,20,43].

*B. plicatilis *belongs to a cryptic species complex [44,45] with two species--*B. manjavacas *and *B. plicatilis *species--that need to be identified by molecular techniques [46] and that coexist in some ponds in Spain [44]. Hence, identification by PCR amplification of the microsatellite marker *Bp1 *[47] was applied, as this marker specifically amplifies for *B. plicatilis *[10]. All the clones coming from HOS, where *B. manjavacas *occurs [48], were identified using this method. In the case of the other three populations where *B. manjavacas *has never been detected, amplification was conducted for a sample of 50 clones in order to confirm its absence.

### Experiment overview

In this study, two cross-mating experiments (F1 experiment and BC experiment) and three sexual generations are involved: P, the parental generation, composed of clones from diapausing (i.e., sexually-produced) eggs isolated from the field; F1, composed of clones produced by crossing P clones in the F1 experiment; and BC, composed of clones produced by crossing P clones and F1 clones (Figure 1, Table 2). The diapausing eggs produced by crossing P clones are termed F1 diapausing eggs, and those produced by crossing P and F1 clones are termed BC diapausing eggs. Therefore, F1 clones are the result of parthenogenetic proliferation from F1 diapausing eggs. F1 clones were used in the experiments with two purposes: (1) to measure fitness components; and (2) to produce individuals for the BC experiment.

**Table 2:** Cross combinations performed in the F1 and BC experiments.

F1 experiments		BC experiments	
**Cross combination**	**Tests**	**Cross combination**	**Tests**

TOS × TOS	a(1), b(1), c(1)	TOS × (TOS × TOS)	d(1), e(1), f(1)
TOS × HOS	a(2)	(TOS × TOS) × TOS	d(1), e(1), f(1)
HOS × TOS	a(2)	TOS × (HOS × TOS)	d(2)
HOS × HOS		TOS × (TOS × HOS)	d(2)
TOS × TUR	b(2)	(HOS × TOS) × TOS	d(2)
TUR × TOS	b(2)	(TOS × HOS) × TOS	d(2)
TUR × TUR		TOS × (TUR × TOS)	e(2)
TOS × CHI	c(2)	TOS × (TOS × TUR)	e(2)
CHI × TOS	c(2)	(TUR × TOS) × TOS	e(2)
CHI × CHI		(TOS × TUR) × TOS	e(2)
		TOS × (CHI × TOS)	f(2)
		TOS × (TOS × CHI)	f(2)
		(CHI × TOS) × TOS	f(2)
		(TOS × CHI) × TOS	f(2)

In the crosses, the first acronym refers to the population (or outcrossed offspring) providing the females. If the acronym is not in parentheses, the corresponding clones were isolated in the field, while terms in parentheses are F1 clones. Comparison tests are indicated by different letters, and the groups compared are indicated by different numbers. Note that 'immigrant × immigrant' crosses performed in F1 were not included in the statistical analyses.

### Foundation and maintenance of the rotifer clones

To obtain the P clones from the field, diapausing eggs morphologically identified as putative *B. plicatilis *eggs were isolated from the sediment samples by the sugar flotation technique described in Gómez & Carvalho [20] with small modifications [18]. Diapausing-egg hatching was induced by individually transferring the eggs into 96-multiwell dishes (Nunc™) containing 150 μl of 6 g L^-1 ^artificial seawater (ASW; Instant Ocean^®^, Aquarium Systems) and incubating the dishes at 25°C under constant illumination (150-170 μmol quanta m^2 ^s^-1^) [18,44,49]. The wells were checked daily for hatchlings up to the fourteenth day. The medium was renewed every other day to prevent fungal and bacterial proliferation [18]. 50 μl of culture medium were added to each well where a newborn female was observed. The rotifer culture medium was a culture of the microalgae *Tetraselmis suecica *grown in f/2 enriched medium [50] at 12 g L^-1 ^ASW (Instant Ocean ^®^, Aquarium Systems). Algae concentration was adjusted to approximately 10^6 ^cells mL^-1 ^using ASW at 12 g L^-1^. After a few days of clonal propagation from the diapausing egg hatchlings, the rotifer clonal cultures were transferred to 1 mL culture medium and maintained at 18°C under constant illumination (PAR: approximately 35 μmol quanta m^2 ^s^-1^). These maintenance conditions were used for clones, cross-mating experiments, and algal cultures and were selected because preliminary experiments showed that they were suitable for obtaining high fertilisation proportions in the crosses. After a few more days of proliferation, the rotifer clones were transferred to a culture volume of ca. 20 mL. Clone cultures were fed weekly by replacing 15 mL of the culture, including rotifers, with fresh culture medium. Sexual reproduction in *Brachionus *depends on population density [51-53], the density effect being rather variable among clones [54,55]. The feeding procedure ensured that all clones would achieve high levels of sexual reproduction between feeding events, so that males and sexual females could be obtained for subsequent cross-mating experiments.

Hatching of F1 and BC diapausing eggs was induced a minimum of one month after they were produced, since it maximizes the hatching proportion, and hatching is rather synchronic [56]. During their storage period, the eggs were kept in darkness at 4°C [18] in 60 g L^-1 ^ASW. The F1 clones used in the BC experiment were founded from F1 diapausing eggs following the procedure described above for P clones.

### Cultures for fitness component measurements

For fitness component measurement in the F1 and BC generations, hatching of the F1 and BC diapausing eggs was induced, and each hatchling was allowed to proliferate for seven days, thus producing a clone. Diapausing egg hatching conditions were similar to the conditions described above for diapausing eggs isolated in the field; differences included: (1) Concentrated algae from a single frozen batch were used for all the cultures in order to minimise variation in food quality [57]. Details on food preparation are in Tortajada et al. [23]. Final concentration was 500,000 cells mL^-1^. (2) As population growth rate was one of the fitness measurements estimated in the F1 and BC clones (see below), diapausing eggs were checked for hatchlings every 12 hours instead of every day, in order to decrease uncertainty about the hatching time. (3) Food was provided when the eggs were transferred to the hatching induction conditions, and hatching was induced at 12 g L^-1 ^salinity; in this way the hatchlings were already in the growing conditions when they were born. (4) The diapausing egg checking period for hatching was extended to 3 weeks. (5) Due to practical constraints, the medium was only renewed once a week during the hatching induction period. (6) Clones were cultured in 30 mL of culture medium. The cultures were allowed to grow for 7 days, and then they were fixed by adding 150 μl of a Lugol's solution to facilitate counting.

### Experimental design and set-up

In order to obtain the F1 generation, two interpopulation and two intrapopulation cross combinations were conducted by combining males and females from TOS, which was regarded as the resident population, and the immigrant source population (HOS, TUR or CHI). This resulted in a total of 10 cross combinations (Table 2). In each cross combination, 40 replicate crosses using different clones were used to obtain the F1 generation, while 36-42 replicate crosses were used to estimate the fertilisation proportion. After obtaining the F1 generation, the F1 clones were backcrossed with P clones from the resident population to obtain the BC generation, with the objective of analysing the subsequent effects of outcrossing (Figure 1, Table 2). Given the expectation that immigrants are rare, backcrosses involving the resident population and F1 immigrant × immigrant offspring were not performed. In each BC combination, 16-20 replicate crosses were performed using different clones. No clone was used in more than one cross.

The cross-mating experiments were performed as follows: virgin, newborn individuals of both sexes were used for the crosses. This was necessary not only to control paternity, but also because: (1) male fertility declines after 8 hours of age [58]; (2) males select young females [59]; and (3) haploid egg fertilisation declines in females over 4 hours of age [58]. In clone cultures where a high number of sexual females were observed, aliquots of approximately 8 ml of rotifer culture were gently shaken in order to detach the eggs from their mothers [59]. The detached eggs exhibiting embryo movement (i.e., in an advanced stage of development) were picked out. These eggs usually hatch in less than 4-5 hours. Eggs with male embryos can easily be distinguished from those harbouring female embryos because the former are smaller in size. Fifty eggs with male embryos from the paternal clone and fifty eggs with female embryos from the maternal clone were transferred to 500 μL of culture medium in a well of a multiwell plate (Iwaki brand, Asahi Techno Glass). This small volume facilitates male-female encounters, but individuals can swim freely, given their small size [60]. After allowing 24 hours for mating, females, which at this point could not be differentiated as sexual or asexual, were isolated individually in wells of a multiwell plate (Nunc™) with 150 μl of culture medium. Note that the number of sexual females used in each replicate cross was variable, due to the impossibility of differentiating between sexual and asexual females *a priori*; moreover, females (and males) to be crossed were isolated as eggs, as explained before. After 2-3 days, females were classified according to the type of egg and offspring they produced as: (1) asexual females (those producing female offspring); (2) unfertilised sexual females (those producing male offspring); or (3) fertilised sexual females (those producing diapausing eggs). Some of the latter females produced several eggs.

In order to estimate fitness components in the F1 generation, 40 diapausing eggs per cross combination were used, each diapausing egg coming from a different cross (i.e., from a different replicate cross of the cross combination). Thus, fitness was estimated on diapausing eggs (and their derived clones) that originated from two independent parental clones. Extra F1 diapausing eggs from each replicate cross were stored, in order to produce the F1 clones needed to perform the backcrosses after their obligate period of diapause.

### Fitness components and data analysis

The following measurements were performed: (1) fertilisation proportion, the proportion of sexual females producing diapausing eggs; (2) diapausing egg-hatching proportion, where an egg was considered hatched when the hatchling had completely emerged from the egg and was able to swim freely; (3) clone viability, where an individual F1 or BC generation clone was considered viable if at least one female was alive on the seventh day of culture, and where, for each cross combination, clone viability was computed as the proportion of the hatched diapausing eggs resulting in viable clones in culture; (4) the initial net growth rate *R*, calculated for each viable F1 or BC generation clone using the equation *N*_*t*_*= N*_*0 *_*R*^*t*^, where *N*_*t *_is the number of females on the seventh day of culture (*t*= 7), and *N*_*0*_= 1; this computation does not assume exponential growth; in fact, growth was affected by at least a density-dependent factor, namely sexual reproduction induction; (5) the proportion of male-producing clones, calculated for each cross combination as the proportion of F1 or BC generation clones among the viable clones where males were observed. While a single measurement of the above traits 1, 2, 3, and 5 was obtained for each cross combination, several *R *values--one per clone within a replicate cross--were obtained. Additionally, the compound measure of fitness 'number of females produced per incubated diapausing egg' was calculated. This parameter was 0 if a diapausing egg did not hatch, or if a clone was not viable by the seventh day, thus it integrated the fitness components 'egg-hatching proportion', 'clone viability' and *R*, and indicated the global effect of outcrossing throughout the rotifer life cycle.

As the measurements 1, 2, 3, and 5 represent binary data, statistical analyses based on generalised linear models assuming a binomial distribution were applied (Table 2), using logit as the link function, and tested with a Chi-square test. For *R*, robust one-way ANOVAs, specifically a robust generalisation of the Box test [61], were performed (Table 2). In all these analyses, the response (measurements 1-5) was modelled as explained by the type of cross. For the F1 experiment the two interpopulation cross-combinations ('immigrant paternal clone × resident maternal clone' and 'immigrant maternal clone × resident paternal clone') were compared with the intrapopulation cross of the resident population. For the BC experiment the four hybrid-resident cross combinations were compared with the two resident-resident cross combination (Table 2). The analysis was applied separately to the three immigrant populations. The data for the number of females produced per incubated diapausing egg were non-independent because they represented a combination of the other measurements; thus, these data were not statistically analysed. In all statistical analyses, two-tailed tests were used because it was difficult to predict the direction of the outcrossing effects due to complex genetic processes [see e.g. [7]]. Additionally, we tested for sex-dependent asymmetries in the fertilisation proportions. A Chi-square test was applied to a 2 × 2 contingency table (columns: 'fertilised sexual females' and 'unfertilised sexual females'; rows: 'paternal population is the immigrant' and 'maternal population is the immigrant'). This test was applied to the three interpopulation crosses at F1 and BC. The statistical analyses were carried out using the R statistical software v. 2.7.2 (R Development Core Team, 2008), using functions included in the WRS package [62] in the case of the robust tests.

## Authors' contributions

All authors participated in the design of the experiments and the statistical analyses and interpretation of data. AMT performed the experiments. All authors jointly wrote the manuscript, and read and approved the final version.

## Supplementary Material

Additional file 1 **Raw data from the cross combinations performed in the F1 experiment**. For each cross combination, number of replicates and the observations used for the estimation of fitness components are shown. Click here for file

Additional file 2 **Raw data from the cross combinations performed in the BC experiment**. For each cross combination, number of replicates and the observations used for the estimation of fitness components are shown. Click here for file
